# The Concept of Radical Responsibility for Non-human Animals

**DOI:** 10.3389/fpsyg.2020.01487

**Published:** 2020-07-24

**Authors:** Dominika Dzwonkowska

**Affiliations:** Institute of Philosophy, Cardinal Stefan Wyszynski University, Warsaw, Poland

**Keywords:** human-animal relations, animal ethics, radical responsibility, moral response, animal use ethics, animal usage

## Introduction

As La Follette said, “we are [.] part of a culture which rather cavalierly uses animals for food, clothes, for research in the development of new drugs, and to determine the safety of household products or cosmetics. And many of these uses require inflicting a great deal of pain on animals” (La Folette, 1989, p. 80). From this point of view, I assume that we should look at non-human animals not from a human-related perspective but from a suffering-related one (Nussbaum, 2004), as suggested by (Bentham, 2000 – first edition 1781). Only then can cruel practices be limited. In this opinion piece, I claim that radical responsibility is conducive as a tool to direct our actions so that they minimize the suffering of non-human animals derived from those actions. Only radical responsibility covers all ranges of animal uses that are present in Western culture—those presented as *explicite* as well as those presented as *implicite* in our daily lives. Thus, I present the concept of responsibility present in previous animal/environmental ethics discussions^1^.

## Moral Responsibility in Ethics

Responsibility for one's actions is one of the most important concepts in ethics, and it is strictly connected to the issue of blameworthiness and praiseworthiness. Moral responsibility has the following formula: “subject S is morally responsible (i.e., blameworthy or praiseworthy) to degree *d* for object O” (Khoury, 2017, p. 2). It involves a three-place predicate that relates to certain subject and object. Historically, many philosophers have focused on individual responsibility. However, after the World War II, some philosophers raised the issue of responsibility as a constructive answer or explanation to what has happened (Jonas, 1987; Levinas, 1987). Others philosophers rather focused on its collective context (Lewis, 1948; Feinberg, 1970; Arendt, 1987). According to (French, 1976, p. 443–444), this situation is similar to the object of our responsibility—it can be an individual action or collective action, i.e., action that can be brought about collectively. In terms of animals, this distinction applies as well.

Apart from the two mentioned distinctions, Khoury (2017, p. 3) also introduced a temporal distinction in the concept of responsibility, namely, synchronic responsibility and diachronic responsibility: “More precisely, synchronic responsibility concerns the extent to which an agent at time t_1_ is responsible for an action that occurs at t_1_. Diachronic responsibility concerns the extent to which an agent at some later time t_2_ is responsible for an action that occurred at t_1_. Synchronic responsibility involves the responsibility of an agent at the time of action, while diachronic responsibility involves the responsibility of an agent at some time after the action occurs” (Khoury, 2017).

## Moral Responsibility and Animals

Throughout the history of Western normative ethics, moral responsibility has applied to relations between human beings. However, the idea of widening the moral circle has for some time been present in animal ethics and environmental ethics in the postulate of an expanding moral circle (Singer, 1981) or, to put it differently, an enlarging *moral consideranda* (Birch, 1993). Both concepts claim that our moral obligations are not limited to human beings only. Thus, whom should we consider? All ecosystems (Leopold, 1949)? All living beings (Taylor, 1986)? All sentient beings (Singer, 1975)? Even though I, in this opinion piece, adopt an ecocentric perspective, I focus only on our relationship with non-human animals from the perspective of our responsibility for them. I put forth the concept of responsibility to overcome the ambiguity—or, as it is called by Francione (2000), moral schizophrenia—that marks our relations with animals. The concept of radical responsibility is proposed to be included in our moral choices, as non-human animals can suffer. It is built on the double theoretical foundation—on Hans Jonas' concept of responsibility for the environment as well as on Nigel Dower's claim of radical responsibility.

Jonas' works (Jonas, 1979, 1982, 1984) outlined and elaborated on the concept of responsibility that appeared for the first time in Georg Picht's publications (Picht, 1969), namely, the idea of responsibility for the environment. Thus, claiming that not only human beings are the subjects of moral responsibility, “Hans Jonas considers all nature as an object of human responsibility” (Mantatov and Mantatova, 2015, p. 1,057). However, animal exploitation is so strongly rooted in western culture that simple responsibility for nature is not enough—we need a radical form of responsibility, much like that found in Dower's writings.

## The Radical Responsibility

Radical responsibility is a form of moral responsibility that pushes our moral obligations further. It states that we are responsible “for the unintended (and often unnoticed) consequences of our actions and our failures to act” (Dower, 1989, p. 18). Radical responsibility is not only related to our actions, but Dower also introduces the concept of an indirect footprint on something that includes responsibility for what others have done for us.

This therefore raises a question of responsibility for when we do not perform a deed ourselves but we let the things being done for us or we do not act to prevent some actions even if they are morally wrong or dubious. Nigel Dower explore this issue; he asks a question about the so-called logic of omission and provides an explanation of it. According to him, “there is one difference between typical cases of killing and typical cases of letting die. Whereas, with killing the death of someone is what is intended, either as an end result or as a necessary means to an end; with “letting die” the death of someone is neither what one aims to achieve—one does not want them to die—nor is it a means to some other end. It is simply an unwanted and often unthought of consequence of pursuing one's other objectives” (Dower, 1983, p. 22). However, in terms of the concept of indirect footprint, we are still responsible for it; the unwanted or unthought consequences of our actions are still in the range of our moral responsibility, and we should act in a way that enables us to escape it. Dower claims that “if it is in our power to prevent something very bad happening without thereby sacrificing anything of moral importance, we ought to do it” (Dower, 2018). Peter Singer also makes a very clear point about not acting when one can do something to prevent the wrong things from happening: “passivity, when people are able to act to prevent evil, is morally wrong” (Singer, 1985, p. 834).

## Radical Responsibility and Non-Human Animals

The human–animal relation is a complex one. It involves all abovementioned forms of moral responsibility, and this could be seen in many cases of animal uses, such as in analyzing animal experimentation. For example, a subject might be the individual and collective; the individual researcher holds a responsibility for his experiments, but he is also a part of bigger community that enabled him to conduct experiments on animals (for example, ethical committees that allow the use of animals, research funding institutions, the whole of the research community that agrees that *in vivo* experiments are an agreeable practice, and even administrative or technical workers that make experimentation possible or are part of it). The object of our responsibility might be individual or collective. To provide compelling evidence, the research must be carried out on a group of animals. Only this will provide profound data. This collective sacrifice of animal lives includes a significant amount of non-human animal suffering (see Singer, 1975). The temporal dimension is also included in animal use, as the researcher is responsible synchronically as well as diachronically for the pain inflicted during experimentation (for example, if a non-human animal is not provided with proper anaesthetization) as well as long-term consequences (for example, if animal is not cared for properly after experimentation or experimentation causes durable consequences that condemn an animal to euthanasia). These are some of the examples of responsibility for animals that have been discussed for quite a long while, at least since Singer's (1975) eye-opening publication.

However, the human–animal relation is far more complex; most of non-human animal uses or exploitation involve a lack of our direct engagement into actions. In most cases involving animal suffering, we are not doers—we might never perform an action that inflicts pain on a non-human animal, and yet we might use a cosmetic product or medical equipment or a drug that has been tested on some sentient being. We might never kill an animal, but eating meat, for example, supports an industry that derive benefits out of it. Thus, we may benefit from these actions indirectly by choosing a good that was produced in a way that involves animal use. Recognition of our moral obligation for indirect footprint and for cases of omission enables us to take a radical responsibility for animals. This is a step further along in our moral approach to non-human animals. Thus, the radical form of moral responsibility for non-human animals invites us to consider every single choice involved in what we eat, buy, or support. It also necessitates active involvement in actions to alleviate animal suffering. It is necessary to avoid passivity and omission that might lead to causing pain to any sentient being. The idea of radical responsibility has been present *implicite* in previous discussions of animal ethics, and this paper is a contribution to conceptualize it and recognize it as a form of moral progress in our relations with non-human animals.

## Author Contributions

The author confirms being the sole contributor of this work and has approved it for publication.

## Conflict of Interest

The author declares that the research was conducted in the absence of any commercial or financial relationships that could be construed as a potential conflict of interest.

## Footnotes

^1^The differences and connections between these two disciplines are explained by Criscuolo and Sueur (2020).

## References

[B1] Arendt H. (1987). Collective responsibility, in Amor Mundi, ed J. W. Brenner (Dordrecht: Martinus Nijhoff Publishers), 50.

[B2] Bentham J. (2000). Introduction to the Principles of Morals and Legislation. Kitchener, ON: Batoche Books.

[B3] Birch T. (1993). Moral considerability and universal consideration. Environ. Ethics 4, 313–332.

[B4] Criscuolo F. Sueur C. (2020). An evolutionary point of view of animal ethics. Front. Psychol. 11:403. 10.3389/fpsyg.2020.00403 32300318 PMC7142228

[B5] Dower N. (1983). World Poverty: Challenge and Response. York: The Ebor Press.

[B6] Dower N. (1989). Ethics and Environmental Responsibility. Aldershot: Gower Publishing Company.

[B7] Dower N. (2018). Global Citizenship: Goals and Means, Lecture delivered on 19.11.2018 at Nocolai Copernicus University in Torun (Poland).

[B8] Feinberg J. (1970). Collective responsibility, in Doing and Deserving: Essays in the Theory of Responsibility, ed J. Feinberg (Princeton, NJ: Princeton University Press), 222–251.

[B9] Francione G. (2000). Animal Rights: Your Child or the Dog?. (Philadelphia, PA: Temple University Press).

[B10] French P. (1976). Senses of blame. Southern J. Philos. 4, 443–452.

[B11] Jonas H. (1979). Das Prinzip Verantwortung: Versuch einer Ethik für die technologische Zivilisation. Frankfurt am Main: Insel-Verlag.

[B12] Jonas H. (1982). The Phenomenon of Life: Toward a Philosophical Biology. Chicago, IL: University of Chicago Press.

[B13] Jonas H. (1984). The Imperative of Responsibility: In Search of Ethics for the Technological Age. Chicago, IL: University of Chicago Press.

[B14] Jonas H. (1987). The concept of god after auschwitz: a Jewish voice. J. Relig. 1, 1–13.

[B15] Khoury A. C. (2017). Individual and Collective Responsibility, in Reflections on Ethics and Responsibility. Essays in Honor of Peter A. French, ed Z. J. Goldberg (Munich: Springer), 1–20.

[B16] La Folette H. (1989). Animal rights and human wrongs, in Ethics and Environmental Responsibility, ed N. Dower (Aldershot: Avebury), 79–90.

[B17] Leopold A. (1949). A Sand County Almanac: And Sketches Here and There. Oxford: Oxford University Press.

[B18] Levinas E. (1987). Time and the Other. Pittsburgh, PA: Duquesne University Press (published in French in 1978).

[B19] Lewis H. D. (1948). Collective responsibility. Philosophy 24, 3–18.

[B20] Mantatov V. Mantatova L. (2015). Philosophical underpinnings of environmental ethics: theory of responsibility by Hans Jonas. Proce. Soc. Behav. Sci. 214, 1055–1061. 10.1016/j.sbspro.2015.11.704

[B21] Nussbaum M. C. (2004). Mill between Aristotle & Bentham. Daedalus 133, 60–68. 10.1162/001152604323049406

[B22] Picht G. (1969). Mut zur Utopie. Die großen Zukunftsaufgaben. Zwölf Vorträge. Munich: Piper-Verlag.

[B23] Singer P. (1975). Animal Liberation. A New Ethics for Our Treatment of Animals. New York, NY: HarperCollins.

[B24] Singer P. (1981). The Expanding Circle: Ethics, Evolution, and Moral Progress. Princeton, NJ: Princeton University Press.

[B25] Singer P. (1985). Famine, affluence, and morality, in Vice & Virtue in Everyday Life, eds Sommers C. Sommers F. (Forth Worth, TX: Harcourt Brace College Publishers), 834–844.

[B26] Taylor P. (1986). Respect for Nature: A Theory of Environmental Ethics. Princeton, NJ: Princeton University Press.

